# Correlation analysis of deep learning methods in S‐ICD screening

**DOI:** 10.1111/anec.13056

**Published:** 2023-03-15

**Authors:** Mohamed ElRefai, Mohamed Abouelasaad, Benedict M. Wiles, Anthony J. Dunn, Stefano Coniglio, Alain B. Zemkoho, John Morgan, Paul R. Roberts

**Affiliations:** ^1^ Cardiac Rhythm Management Research Department University Hospital Southampton NHS Foundation Trust Southampton UK; ^2^ Faculty of Medicine University of Southampton Southampton UK; ^3^ Aberdeen Royal Infirmary Scotland UK; ^4^ School of Mathematical Sciences University of Southampton UK; ^5^ Department of Economics University of Bergamo Bergamo Italy

**Keywords:** deep learning tools, screening, subcutaneous implantable cardiac defibrillators

## Abstract

**Background:**

Machine learning methods are used in the classification of various cardiovascular diseases through ECG data analysis. The concept of varying subcutaneous implantable cardiac defibrillator (S‐ICD) eligibility, owing to the dynamicity of ECG signals, has been introduced before. There are practical limitations to acquiring longer durations of ECG signals for S‐ICD screening. This study explored the potential use of deep learning methods in S‐ICD screening.

**Methods:**

This was a retrospective study. A deep learning tool was used to provide descriptive analysis of the T:R ratios over 24 h recordings of S‐ICD vectors. Spearman's rank correlation test was used to compare the results statistically to those of a “gold standard” S‐ICD simulator.

**Results:**

A total of 14 patients (mean age: 63.7 ± 5.2 years, 71.4% male) were recruited and 28 vectors were analyzed. Mean T:R, standard deviation of T:R, and favorable ratio time (FVR)—a new concept introduced in this study—for all vectors combined were 0.21 ± 0.11, 0.08 ± 0.04, and 79 ± 30%, respectively. There were statistically significant strong correlations between the outcomes of our novel tool and the S‐ICD simulator (*p* < .001).

**Conclusion:**

Deep learning methods could provide a practical software solution to analyze data acquired for longer durations than current S‐ICD screening practices. This could help select patients better suited for S‐ICD therapy as well as guide vector selection in S‐ICD eligible patients. Further work is needed before this could be translated into clinical practice.

## Graphical abstract

Favorable ratio time as an outcome of our deep learning tool is plotted on the *x*‐axis against the eligible vector time as the outcome of the “gold standard” S‐ICD simulator, plotted on the *y*‐axis. Spearman's rank test showed strong correlation.

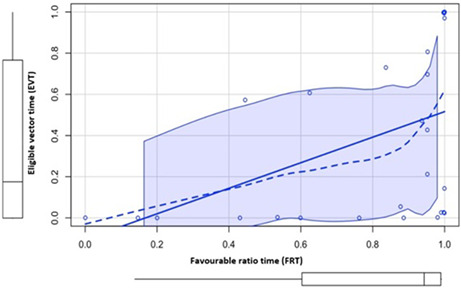

## 1 INTRODUCTION

The subcutaneous implantable cardiac defibrillator (S‐ICD) is an established totally avascular alternative to the traditional transvenous defibrillators (TV‐ICDs). S‐ICDs offer defibrillation therapy while avoiding lead‐related complications associated with traditional ICDs (Lambiase, 2022). However, the Achilles heel of the S‐ICD to date remains the relatively high rate of inappropriate shocks when compared with conventional TV‐ICDs. T‐wave oversensing (TWO) is still the most common cause of inappropriate shock delivery in S‐ICDs (Knops et al., 2020).

Appropriate functioning of the S‐ICD relies on the presence of vectors with suitable ECG morphology. As such, not all patients are eligible for S‐ICDs, and pre‐implant ECG screening is performed on all potential candidates to ensure they have at least one vector that meets the screening criteria (Francia et al., 2018; Groh et al., 2014; Olde Nordkamp et al., 2014; Randles et al., 2014; Rudic et al., 2018). Surface ECGs of few seconds duration done in multiple postures are used as surrogates for the three standard S‐ICD vectors. These are assessed via an automated screening tool built‐into an S‐ICD programmer to determine eligibility (Rudic et al., 2018). A major predictor of eligibility is the T:R ratio. ECG signals sensed by at least one vector needs to pass screening in at least two postural positions for the patient to be deemed eligible. Unfortunately, despite this screening process, TWO remains the commonest cause of inappropriate shock therapies in S‐ICD patients (Kamp & Al‐Khatib, 2019). This is significant as inappropriate shock therapies can have detrimental effects on the quality of life, psychological well‐being, can result in the induction of ventricular arrhythmias and increase all‐cause mortality (Daubert et al., 2008).

T‐wave morphology is dynamic and can alter with position, exercise, electrolyte disturbance, progression of myocardial diseases, and changes in autonomic function (Al‐Zaiti et al., 2011; Assanelli et al., 2013; Hasan et al., 2012; Madias et al., 2001; Mayuga & Fouad‐Tarazi, 2007). This can provide an explanation for the occurrence of TWO events in vectors with ECG signals that originally passed the S‐ICD screening. It is important to highlight that not all TWO events result in inappropriate shocks. If the TWO episode is not sustained long enough to result in capacitator charging, it will pass unmarked, and no record of the event is made. This is because an S‐ICD is only programmed to store episodes of tachycardia that result in capacitor charging. This preserves both battery life and memory capacity of the system. Therefore, the true incidence of TWO in the S‐ICD population is not known.

The concept of the potential varying of S‐ICD vectors eligibility over time was previously presented in a published study by Wiles et al. (Wiles et al., 2021) The study demonstrated that the vector score which determines S‐ICD eligibility is dynamic in real‐life ICD population. For that study, an S‐ICD simulator provided by the device manufacturer was utilized for vector assessment. The clinical significance of this dynamicity has not yet been evaluated. However, it sheds light on the possibility that acquiring screening data over a much longer period than for conventional screening can enable more reliable and descriptive screening of the ECG signals sensed by the S‐ICD vectors and can aid patient and vector selection in S‐ICD candidates. The ultimate goal would be reducing the risk of TWO and inappropriate shocks.

The authors of this article have also previously introduced a novel deep learning‐based screening tool, which provides a detailed descriptive analysis of the behavior of T:R ratios from an S‐ICD perspective could be obtained (Dunn et al., 2021). We theorize that this tool can guide patient selection as well as vector selection in S‐ICD recipients. The aim of this study was to clinically apply this tool to screen a cohort of ICD patients for S‐ICD vectors eligibility and assess our findings against a “gold standard”—an S‐ICD simulator.

## 2 METHODS

This is a retrospective correlation study. In our previous study Wiles et al. (2021), adult ICD (transvenous and S‐ICD) patients were asked to wear Holter monitors for 24 h to record ECG signals corresponding to their S‐ICD vectors. Then, these ECG recordings were analyzed using an S‐ICD simulator to assess the vector scores automatically at regular intervals. Mean vector scores were also analyzed and a new concept—Eligible Vector Time (EVT), representing the percentage of all the screening assessments with passing vector scores—was introduced in the study. The study concluded that the S‐ICD vectors eligibility in an ICD population is dynamic (Wiles et al., 2021).

The same 24 h Holter recordings, sampling rate 500/s, from our previous study were first downloaded. Then, the recordings were analyzed by our deep learning tool. First, the data is split into 10 s segments. Then, phase space reconstruction (PSR) was utilized to convert the ECG signal into compressed 32 x 32 pixel PSR images, one image for each 10 s worth of ECG data.

**FIGURE 1:**
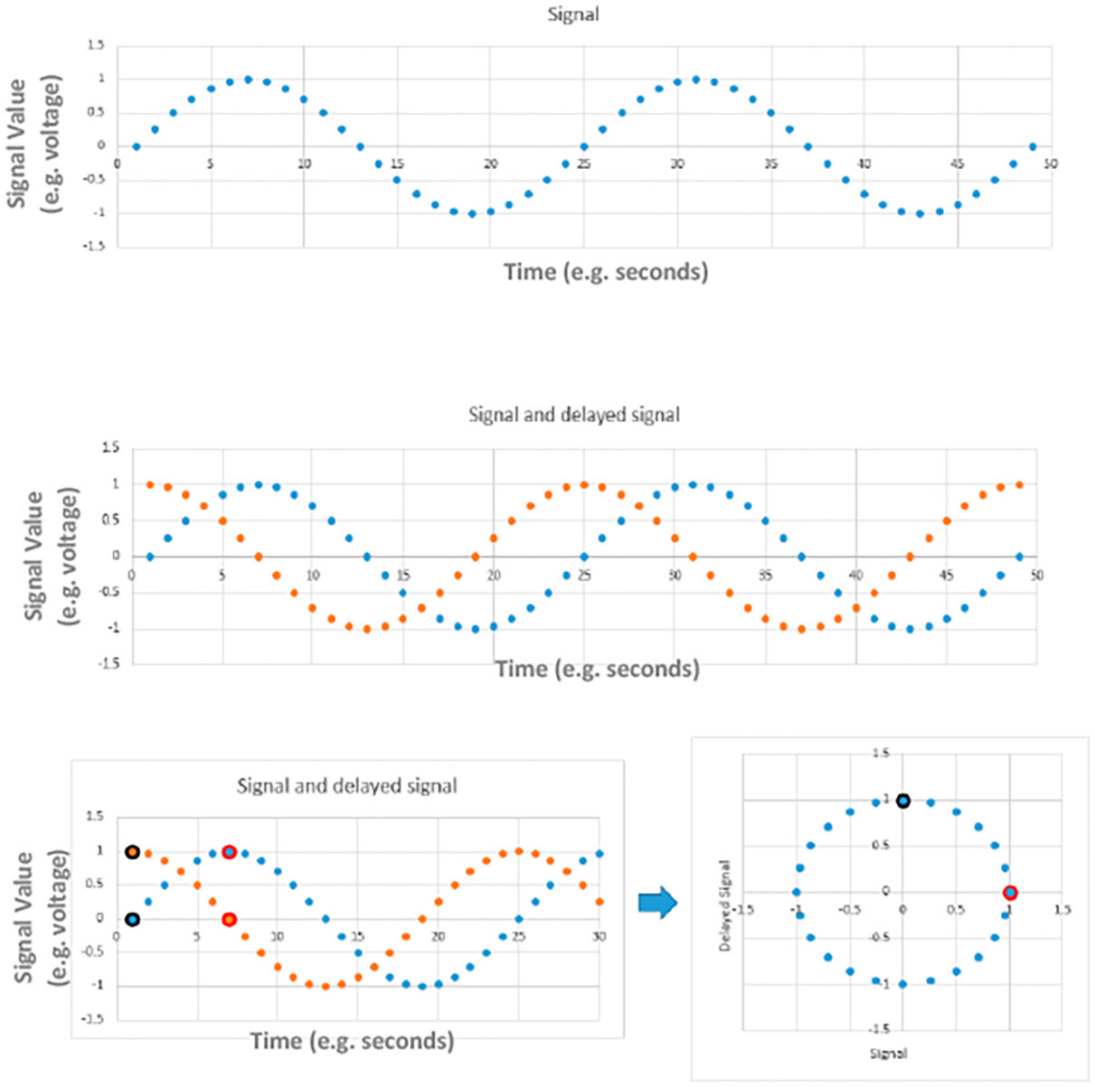
Illustrates how a phase space reconstruction (PSR) image of the sine wave is created. As the sine wave has a repeated behavior, the sine wave signal could have any length and the phase space points generated from this signal would continuously trace and retrace the resulting circle figure which represents the PSR image of the sine wave.

Phase space reconstruction is a popular technique in waveform analysis for representing nonlinear characteristics of time series set of data using delay maps. Typically, when a signal is examined, it is plotted against time. To construct the PSR of the signal, a copy of the signal, which is delayed by a given amount of time, is first created. Then, the original signal is plotted against the delayed signal. At each time increment, a single point in the phase space reconstruction is created. Each point has an *x*‐axis value equal to the value of the original signal at that time increment and a *y*‐axis value equal to the value of the delayed signal at that time increment. By removing the time axis from this plot, the repetitive behaviors of the signal can be seen (Rocha et al., 2008). An example of how a signal can be transformed into its PSR image is illustrated in Figure 1.

Several techniques have been used before to analyze PSR. Box counting, where simply the image or the phase space is divided up into a grid and the number of boxes occupied by the image is counted. Also, measuring the area covered by the PSR plot and calculating summary statistics for rows and columns of the PSR matrix.

In the algorithm used here, a multilayered Convoluted Neural Network (CNN) is trained to automatically determine the optimal features to extract from the transformed ECG 32 × 32‐pixel PSR images. This CNN is made up of a number of feature extraction blocks, followed by a regression block. The outputs from the preceding feature extraction blocks are flattened to a 1D vector and fed into a series of fully connected (dense) layers of neurons to arrive at the final regression output: the T:R ratio.

**FIGURE 2:**
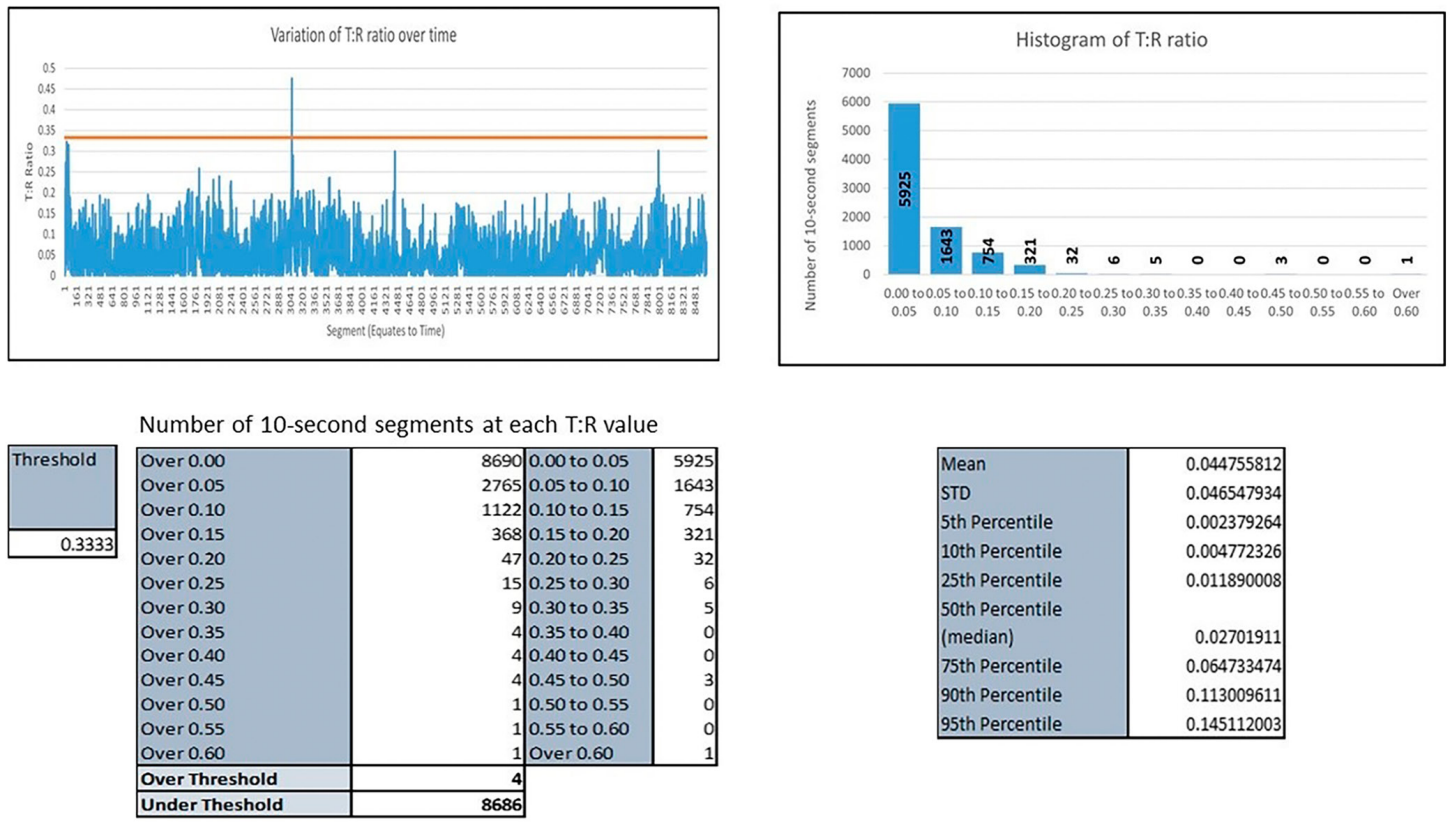
One example of the results of vector analysis produced by our tool; This is the T: R analysis of the alternate S‐ICD vector of patient 03.

The end result is a plot showing the mean T:R, standard deviation (SD), and the number of 10 s segments that has T:R above a predetermined threshold for the ECG signals sensed by each lead/S‐ICD vector over the recorded period. See Figure 2. For further details on the algorithm development, refer to our previously published work by Dunn et al. (Dunn et al., 2021)

The time that a T:R ratio of a vector was deemed favorable (below the eligibility threshold) was calculated as a percentage of the whole recording (=number of 10 s segments with T:R below eligibility threshold/total number of 10 s segments in the recording × 100). For this article, this was labeled as favorable ratio time or FRT.

### 2.1 Statistical methodology

Data analysis was done using RStudio 1.4.1106 running R 4.0.5. Continuous data were presented as mean ± SD. The distribution of the data was checked using normality tests and plots, and histograms. The correlation was checked using Spearman's rank coefficient among variables that fit interval, ordinal or ratio scale, and in paired observations with monotonic relation assumption.

To correlate the outcome of our deep learning tool with that of the S‐ICD simulator, the following were compared statistically: (mean vector score + standard deviation of the vector score) and (mean T:R + standard deviation of the T: R, mean T:R + standard deviation of T:R) and EVT, and finally FRT and EVT.

## 3 RESULTS

### 3.1 Patients' demographics

A total of 14 patients (mean age: 63.7 ± 5.2 years, 71.4% male) were recruited in the original study. The primary and alternate vectors for each of the patients, amounting to a total of 28 vectors were analyzed. A total of 13 (92.9%) patients had transvenous ICDs. There was a high prevalence of ischemic heart disease (42.9%) and severe (ejection fraction <35% on echocardiogram) LV dysfunction (28.6%) in the recruited cohort. The main indication for ICD therapy was secondary prevention (71.4%). See Table 1 for detailed patients' demographics.

**TABLE 1:** Patient demographics.

		*n* = 14	(%)
Demographics	Mean age (years ± 95% Cl)	63.7 (±5.2)	
	Male	10	71.4
Device	Primary prevention	4	14
	Secondary prevention	10	71.4
	Transvenous ICD	13	92.9
	Subcutaneous ICD	1	7.1
Comorbidities	Ischemic heart disease	6	42.9
	Severe LV systolic dysfunction	4	28.6
	Previous atrial arrhythmia	3	23.1
	Hypertension	3	23.1
	Airway disease	3	23.1
	Diabetes	2	14.3
	Valve disease (>mild) or valve surgery	2	14.3
	Previous CABG	2	14.3
	Cerebrovascular disease	1	7.1
	Peripheral vascular disease	1	7.1
	eGFR < 60 mL/min/1.73^2^ (*n* = 10)	1	10
	eGFR < 30 mL/min/1.73^2^ (*n* = 10)	1	10

Abbreviations: CABG, coronary artery bypass graft surgery; eGFR, estimated glomerular filtration rate; LV, left ventricle.

### 3.2 T:R assessment

Mean T:R was lower in the primary vectors when compared to the alternate vectors (0.20 ± 0.06 vs. 0.22 ± 0.06, *p* = .30). Standard deviation of the T:R (a representation of dynamicity) was also lower in the primary vectors (0.07 ± 0.02 vs. 0.09 ± 0.02, *p* = .11). This has translated to a higher favorable ratio time (FRT) in the primary vectors when compared to the alternate vectors (87.1 ± 14.13 vs. 70.4 ± 16.16%, *p* = .07).

Mean T:R for all the 28 vectors combined was 0.21 ± 0.11, standard deviation for all the 28 vectors combined was 0.08 ± 0.04, and the FRT for all the vectors combined was 79 ± 30%. For individual assessment of each vector, see Table 2.

**TABLE 2:** Results of T: R assessment using the deep learning tool.

Study ID	Vector	Mean T:R	T:R standard deviation	T:R segments over threshold	T:R segments below threshold	Favorable ratio time (FRT) (%)
01	Alternate	0.274	0.078	2045	6565	76.25
01	Primary	0.057	0.064	28	8582	99.67
02	Alternate	0.075	0.062	5	8785	99.94
02	Primary	0.175	0.064	84	8706	99.04
03	Alternate	0.045	0.047	4	8686	99.95
03	Primary	0.110	0.107	411	8279	95.27
04	Alternate	0.199	0.138	980	7630	88.62
04	Primary	0.164	0.107	545	8065	93.67
05	Alternate	0.113	0.076	20	8590	99.77
05	Primary	0.102	0.046	10	8600	99.88
06	Alternate	0.363	0.180	4902	3708	43.07
06	Primary	0.187	0.098	414	8196	95.19
07	Alternate	0.297	0.139	4003	4607	53.51
07	Primary	0.479	0.026	8605	5	0.06
08	Alternate	0.382	0.059	6949	1740	20.03
08	Primary	0.311	0.032	1420	7269	83.66
09	Alternate	0.338	0.023	4778	3832	44.51
09	Primary	0.168	0.031	10	8600	99.88
10	Alternate	0.048	0.040	0	8610	100
10	Primary	0.153	0.049	2	8608	99.98
11	Alternate	0.260	0.139	3452	5158	59.91
11	Primary	0.203	0.088	410	8200	95.24
12	Alternate	0.381	0.060	7346	1264	14.68
12	Primary	0.195	0.027	0	8610	100
13	Alternate	0.203	0.107	944	6760	87.75
13	Primary	0.340	0.107	2891	4813	62.47
14	Alternate	0.142	0.070	168	8442	98.05
14	Primary	0.143	0.105	413	8197	95.20
Mean	Primary	0.20 ± 0.06 (95% CI)	0.07 ± 0.02 (95% CI)			87.1 ± 14.13 (95% CI)
Mean	Alternate	0.22 ± 0.06 (95% CI)	0.09 ± 0.02 (95% CI)			70.4 ± 16.16 (95% CI)
Mean	Combined	0.21 ± 0.11(95% Cl)	0.08 ± 0.04(95% Cl)			79 ± 30 (95% Cl)

### 3.3 Correlation

Mean vector score was higher in the primary vectors when compared to the alternate vectors (412.6 ± 191 vs. 105.6 ± 139.2, *p* = .008). Standard deviation of vector scores was lower (i.e., more stable vector scores) in the primary vectors when compared to the alternate vectors (95.23 ± 76.17 vs. 160.56 ± 60.73, *p* = .10), and the EVT was significantly higher in the primary vectors when compared with the alternate vectors (64.55 ± 19.07 vs. 13.05 ± 15.34%, *p* < .001). Mean vector score for all the vectors combined was 259.09 ± 129.60 (95% CI), the standard deviation of the vector score for all the vectors combined was 127.89 ± 49.36 (95% CI), and the EVT for all the vectors combined was 38.80 ± 15.45 (95% CI).

**FIGURE 3:**
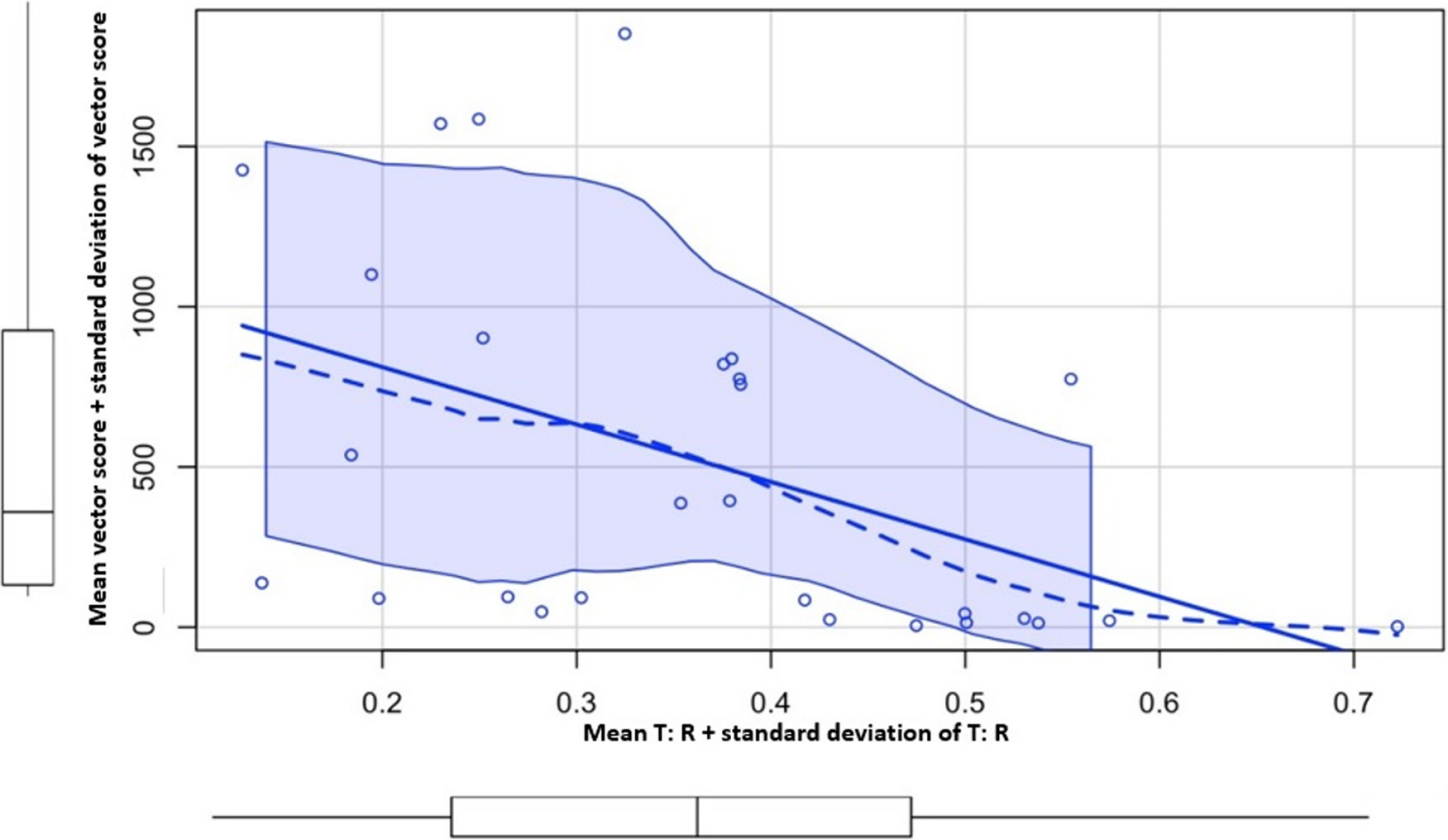
Mean T:R ratio + standard deviation of T:R (*x*‐axis) in correlation with mean vector score + standard deviation of mean vector score (*y*‐axis) using Spearman's rank correlation test. Rho = 0.636 (*p* < .001) denoting strong correlation.

**FIGURE 4:**
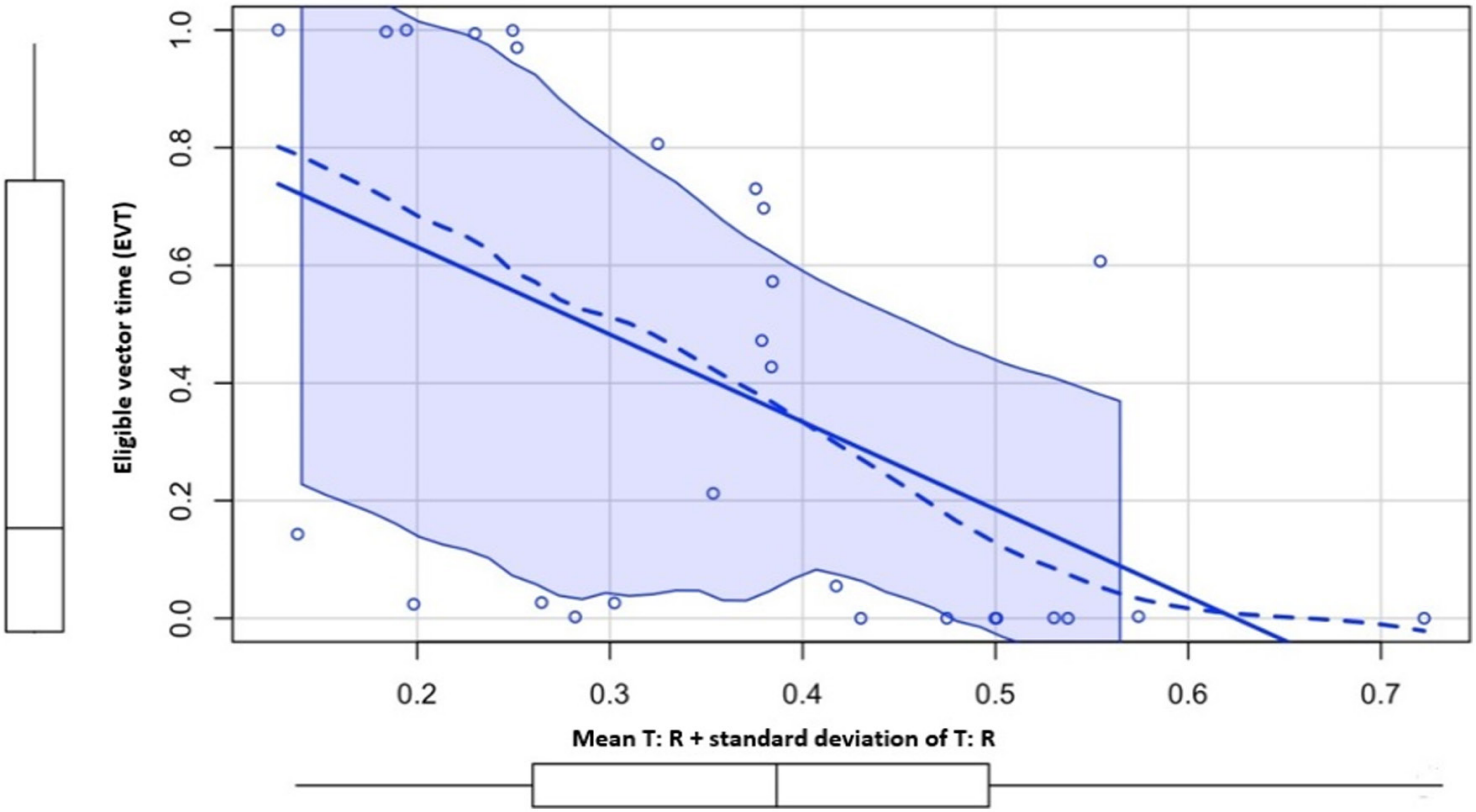
Mean T:R ratio + standard deviation of T:R (*x*‐axis) in correlation with eligible vector time (EVT; *y*‐axis) using Spearman's rank correlation test. Rho = 0.668 (*p* < .001) denoting strong correlation.

**FIGURE 5:**
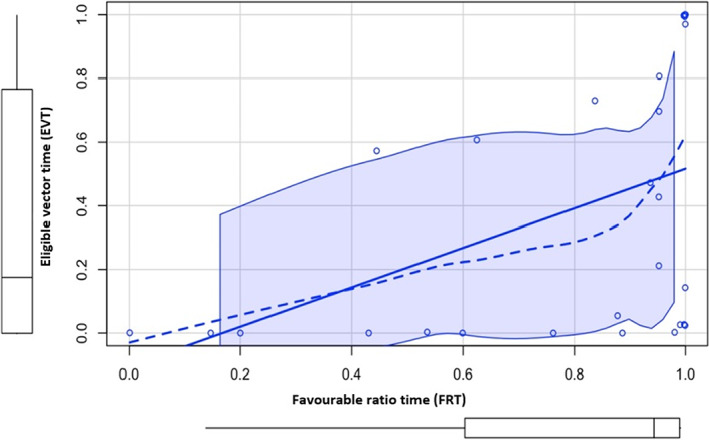
Favorable ratio time (*x*‐axis) in correlation with eligible vector time (EVT; *y*‐axis) using Spearman's rank correlation test. Rho = 0.652 (*p* < .001) denoting strong correlation.

There were statistically significant strong correlations between the outcomes of the proposed tool and the S‐ICD simulator; Mean T:R ratio + standard deviation of T: R correlated strongly with mean vector score + standard deviation of mean vector score, Rho = 0.636 (*p* < .001). Mean T:R ratio + standard deviation of T:R in correlated strongly with eligible vector time (EVT), Rho = 0.668 (*p* < .001). Favorable ratio time also correlated with eligible vector time (EVT), Rho = 0.652 (*p* < .001). See Table 3 and Figures 3, 4, 5 for detailed results.

**TABLE 3:** Outcome of the deep learning tool versus the outcome of the S‐ICD simulator.

Study ID	Vector	Deep learning tool			S‐ICD simulator		
		Mean T:R	T:R standard deviation	Favorable ratio time (FRT) (%)	Mean vector scores	Vector scores standard deviation	Eligible vector time (EVT) (%)
01	Alternate	0.274	0.078	76.25	4.6	9.7	0
01	Primary	0.057	0.064	99.67	385.0	76.2	100
02	Alternate	0.075	0.062	99.94	40.9	24.5	2.4
02	Primary	0.175	0.064	99.04	25.6	33.2	2.62
03	Alternate	0.045	0.047	99.95	57.0	40.6	14.32
03	Primary	0.110	0.107	95.27	807.5	522.1	81
04	Alternate	0.199	0.138	88.62	1.0	2.0	0
04	Primary	0.164	0.107	93.67	120.0	137.2	47
05	Alternate	0.113	0.076	99.77	40.7	27.0	2.68
05	Primary	0.102	0.046	99.88	536.6	282.0	100
06	Alternate	0.363	0.180	43.07	0.2	0.9	0
06	Primary	0.187	0.098	95.19	201.0	286.9	43
07	Alternate	0.297	0.139	53.51	5.1	7.7	0.29
07	Primary	0.479	0.026	0.06	3.9	11.8	0.07
08	Alternate	0.382	0.059	20.03	9.6	16.6	0
08	Primary	0.311	0.032	83.66	386.1	217.3	73
09	Alternate	0.338	0.023	44.51	296.5	230.5	57.25
09	Primary	0.168	0.031	99.88	1046.8	262.0	99
10	Alternate	0.048	0.040	100	989.1	218.5	100
10	Primary	0.153	0.049	99.98	453.7	224.1	97
11	Alternate	0.260	0.139	59.91	3.1	4.9	0
11	Primary	0.203	0.088	95.24	314.1	261.8	79
12	Alternate	0.381	0.060	14.68	4.5	4.7	0
12	Primary	0.195	0.027	100	1161.9	211.7	100
13	Alternate	0.203	0.107	87.75	17.7	33.5	5.48
13	Primary	0.340	0.107	62.47	247.3	263.4	61
14	Alternate	0.142	0.070	98.05	7.9	20.2	0.22
14	Primary	0.143	0.105	95.20	87.1	150.0	21
Mean	Primary	0.20 ± 0.06 (95% CI)	0.07 ± 0.02 (95% CI)	87.1 ± 14.13 (95% CI)	412.6 ± 191 (95%CI)	95.23 ± 76.17 (95%CI)	64.55 ± 19.07 (95%CI)
Mean	Alternate	0.22 ± 0.06 (95% CI)	0.09 ± 0.02 (95% CI)	70.4 ± 16.16 (95% CI)	105.6 ± 139.2 (95% CI)	160.56 ± 60.73 (95% CI)	13.05 ± 15.34 (95% CI)
Mean	Combined	0.21 ± 0.11 (95% Cl)	0.08 ± 0.04 (95% Cl)	79% ± 30 (95% Cl)	259.09 ± 129.60 (95% CI)	127.89 ± 49.36 (95% CI)	38.80 ± 15.45 (95% CI)

## 4 DISCUSSION

### 4.1 T:R ratio

The S‐ICD has three ECG signal sensing vectors, each with their own T:R ratio. A major predictor of eligibility during the ECG signal screening of a vector is the T:R ratio (Francia et al., 2018; Maurizi et al., 2016; Olde Nordkamp et al., 2014; Srinivasan et al., 2017). Large T:R ratios are unacceptable because if the T wave sits above the S‐ICD sensitivity level, this can result in double counting whereby a T wave following a QRS is interpreted as another R wave. This “double counting” can result in inappropriate diagnosis of ventricular tachycardia and subsequently activation of shock therapy. Despite the pre‐implant screening, the commonest cause of inappropriate shocks in the S‐ICD population remains TWOS (Aydin et al., 2012; Bardy et al., 2010; Burke et al., 2015; Dabiri Abkenari et al., 2011; Jarman et al., 2012; Olde Nordkamp et al., 2012). It is important to note that different factors such as changes in posture, heart rate, electrolytes concentrations, body weight, fluid shifts, and lung congestion can cause detectable dynamic changes on surface ECG recordings (Al‐Zaiti et al., 2011; Assanelli et al., 2013; Hasan et al., 2012; Madias, 2005; Madias et al., 2001; Walker et al., 2003). Subsequently, the T:R ratio is not fixed in the same individual. This could provide a potential explanation for the occurrence of TWO in S‐ICD vectors that previously passed S‐ICD screening.

Because of the crucial role of the T:R ratio in the sensing mechanism of the S‐ICD and its subsequent determination of S‐ICD eligibility and TWO events, it was chosen specifically as the parameter to be tracked and analyzed by our novel tool. A T:R ratio eligibility cutoff ratio of 1:3 was chosen based on the manual S‐ICD screening tool following the manufacturer's guidelines (Randles et al., 2014), although the manual screening method is now highly replaced with automatic screening methods, as they follow the same principles.

The concept of integrating deep learning methods in clinical practice is not new. Machine learning methods are already being used in the classification and the prediction of various cardiovascular diseases through ECG data analysis (Fan et al., 2018; IEEE Conference Publication|IEEE Xplore, 2021; Kiranyaz et al., 2016; Lih et al., 2020; Pourbabaee et al., 2018; Roberts, 2001; Rocha et al., 2008; Vemishetty et al., 2019; Zhang et al., 2020). Convolutional neural networks (CNNs) have been used before in ECG analysis for classifying heart attacks, and arrhythmias as well as for predicting blood pressure (Fan et al., 2018; Kiranyaz et al., 2016; Lih et al., 2020; Liu et al., 2018; Miao et al., 2020; Pourbabaee et al., 2018; Sangaiah et al., 2020; Zhang et al., 2020). However, to the best of our knowledge, integrating deep learning tools to predict S‐ICD eligibility has not been reported before.

### 4.2 Practical considerations

Our previous work Wiles et al. (2021) has utilized a S‐ICD simulator‐ provided by the S‐ICD manufacturer—in order to analyze the vector score over the 24 h recordings in real‐time, that is, it took 24 h for the S‐ICD simulator to analyze a 24 h ECG recording for one vector. As the S‐ICD simulator essentially replicates the sensing mechanism of a S‐ICD in real‐time, it can be considered as a “gold standard” for evaluating various durations of ECG signals sensed by S‐ICD vectors. Our study demonstrated that the outcomes of our novel tool correlate strongly with those of the “gold standard” S‐ICD simulator, aside from being time‐efficient. The simulator—aside from being not readily available—runs in real time and analyses the ECG signals sensed by the S‐ICD vectors consecutively, which can be a time‐consuming process, particularly if it is required to analyze recordings of even longer durations. Our tool can provide detailed descriptive analysis of the T:R ratios simultaneously for the ECG signals sensed by all the vectors across the recordings within a few minutes without compromising on accuracy.

Acquiring screening data for eligibility in S‐ICD candidates over a longer period than for conventional screening practices seems like a reasonable approach to minimize the effect of the—at least theoretical—dynamicity of S‐ICD eligibility. However, this approach increases the burden of data analysis required to assess S‐ICD eligibility. Our tool represents a practical software solution that could provide detailed data analysis within minutes; thus, facilitating informed decision‐making and could guide patient selection as well as vector selection in S‐ICD candidates.

While the R:T ratio rather than the T:R ratio is more common in literature, the reason for choosing T:R ratio for this work can be attributed to the deep learning algorithm used for data analysis; as the T‐wave amplitude approaches 0, very small changes in the amplitude can result in extreme changes in the R:T ratio (but not T:R ratio). This massive variation in R:T ratio makes it inappropriate for use as a label in the algorithm, and for this reason, the T:R ratio is used instead (Dunn et al., 2021).

The results of this study denote overall more favorable ratios in the primary vectors on average. However, this might not be true for every individual patient. This highlights the importance of individualizing S‐ICD screenings and tailoring the device programming for each patient.

There are some limitations to our study; first, the relatively low number of ECG signals were analyzed in this study. Second, only the ECG signals sensed by the primary and alternate vectors were available for analysis, because the Holter that was used to collect the S‐ICD vectors was limited to recording only two simultaneous channels. Also, the S‐ICD simulator analyzed the data at 1 min intervals, while our novel tool provided analysis of the T:R ratios at 10 s intervals. In addition, the role of the SMART PASS algorithm that could help differentiate between R and T waves based on other characteristics rather than just their amplitudes, was not considered in this study. We propose that our algorithm is to be used as a supplement and not a replacement of the SMART pass algorithm, that is, ECG signals are processed/filtered first through the SMART pass algorithm, and then, analyzed using our algorithm as an additional step.

Theoretically, our deep learning tool could be potentially used to predict the risk of TWO events and allow informed decisions to be made by the physicians and patients alike prior to committing to S‐ICD therapy. The tool could also be used to guide vector selection in S‐ICD eligible patients. However, further work is needed before it is possible to apply our tool to clinical practice. A prospective study, with a larger number of recruited patients, and a more controlled data protocol, is needed to assess the effect of different physiological states, such as exercise, sudden changes in position or mental stress on the ECG recordings, and subsequently, the outcome of our deep learning tool. In addition, our algorithm needs to be tested on ECG signals, acquired from a larger number of patients, or simulated patients, that are processed with the widely utilized SMART PASS algorithm, to assess if our algorithm could improve upon the current method of sensing vector selection.

## 5 CONCLUSION

T:R ratio—a crucial element in the S‐ICD sensing mechanism and a major determinant of S‐ICD eligibility—is dynamic in “real‐life” ICD patients. Deep learning methods could provide reliable and time‐efficient analysis of T:R ratios. This could help with the S‐ICD screening process as well as guide vector selection in S‐ICD eligible patients. Prospective studies with larger cohorts of recruited patients are needed before the findings from our study could be translated into clinical practice.

## AUTHOR CONTRIBUTIONS

M. ElRefai involved in conceptualization, writing, data curation, analysis, and review and editing. M. Abouelasaad involved in data curation and analysis. B. Wiles involved in conceptualization, and review and editing. A. Dunn, S. Coniglio, and A. Zemkoho involved in data analysis and review. J. Morgan involved in review and editing. P. Roberts involved in conceptualization, data analysis, and review and editing.

## CONFLICT OF INTEREST STATEMENT

Dr. Mohamed ElRefai received unrestricted research grant from Boston Scientific. Benedict Wiles received unrestricted research grant and consultancy fees from Boston Scientific. The work of Anthony J. Dunn is jointly funded by Decision Analysis Services Ltd and EPSRC through the Studentship with Reference EP/R513325/1. Professor John Morgan is a Medical Director at Boston Scientific. Paul Roberts receives consultancy fees from Boston Scientific and Medtronic. Other authors have no conflicts of interests to declare.

## ETHICS STATEMENT

This is a retrospective correlation study. National code on clinical trials has declared that ethics approval is not necessary for real retrospective studies.

## Data Availability

The data that support the findings of this study are available from the corresponding author upon reasonable request

## References

[anec13056-bib-0001] Al‐Zaiti, S. S. , Runco, K. N. , & Carey, M. G. (2011). Increased T wave complexity can indicate subclinical myocardial ischemia in asymptomatic adults. Journal of Electrocardiology, 44(6), 684–688. 10.1016/j.jelectrocard.2011.07.017 21924433 PMC3200448

[anec13056-bib-0002] Assanelli, D. , Di Castelnuovo, A. , Rago, L. , Badilini, F. , Vinetti, G. , Gianfagna, F. , Salvetti, M. , Zito, F. , Donati, M. B. , De Gaetano, G. , & Iacoviello, L. (2013). T‐wave axis deviation and left ventricular hypertrophy interaction in diabetes and hypertension. Journal of Electrocardiology, 46(6), 487–491. 24011993 10.1016/j.jelectrocard.2013.08.002

[anec13056-bib-0003] Aydin, A. , Hartel, F. , Schluter, M. , Butter, C. , Köbe, J. , Seifert, M. , Gosau, N. , Hoffmann, B. A. , Hoffmann, M. , Vettorazzi, E. , Wilke, I. , Wegscheider, K. , Reichenspurner, H. C. , Eckardt, L. , Steven, D. , & Willems, S. (2012). Shock efficacy of subcutaneous implantable cardioverter‐defibrillator for prevention of sudden cardiac death: Initial multicenter experience. Circulation: Arrhythmia and Electrophysiology, 5, 913–919. 22923274 10.1161/CIRCEP.112.973339

[anec13056-bib-0004] Bardy, G. H. , Smith, W. M. , Hood, M. A. , Crozier, I. G. , Melton, I. C. , Jordaens, L. , Theuns, D. , Park, R. E. , Wright, D. J. , Connelly, D. T. , Fynn, S. P. , Murgatroyd, F. D. , Sperzel, J. , Neuzner, J. , Spitzer, S. G. , Ardashev, A. V. , Oduro, A. , Boersma, L. , Maass, A. H. , … Grace, A. A. (2010). An entirely subcutaneous implantable cardioverter‐defibrillator. New England Journal of Medicine, 363, 36–44. 20463331 10.1056/NEJMoa0909545

[anec13056-bib-0005] Burke, M. C. , Gold, M. R. , Knight, B. P. , Barr, C. S. , Theuns, D. A. M. J. , Boersma, L. V. A. , Knops, R. E. , Weiss, R. , Leon, A. R. , Herre, J. M. , Husby, M. , Stein, K. M. , & Lambiase, P. D. (2015). Safety and efficacy of the totally subcutaneous implantable defibrillator: 2‐year results from a pooled analysis of the IDE study and EFFORTLESS registry. Journal of the American College of Cardiology, 65, 1605–1615. 25908064 10.1016/j.jacc.2015.02.047

[anec13056-bib-0006] IEEE Conference Publication | IEEE Xplore . (2021). Classification methodology of CVD with localized feature analysis using Phase Space Reconstruction targeting personalized remote health monitoring . https://ieeexplore.ieee.org/abstract/document/7868773.

[anec13056-bib-0007] Dabiri Abkenari, L. , Theuns, D. A. , Valk, S. D. , Van Belle, Y. , de Groot, N. M. , Haitsma, D. , Muskens‐Heemskerk, A. , Szili‐Torok, T. , & Jordaens, L. (2011). Clinical experience with a novel subcutaneous implantable defibrillator system in a single center. Clinical Research in Cardiology, 100, 737–744. 21416191 10.1007/s00392-011-0303-6 PMC3167040

[anec13056-bib-0008] Daubert, J. P. , Zareba, W. , Cannom, D. S. , McNitt, S. , Rosero, S. Z. , Wang, P. , Schuger, C. , Steinberg, J. S. , Higgins, S. L. , Wilber, D. J. , Klein, H. , Andrews, M. L. , Jackson Hall, W. , Moss, A. J. , & MADIT II Investigators . (2008). Inappropriate implantable cardioverter‐defibrillator shocks in MADIT II: Frequency, mechanisms, predictors and survival impact. Journal of the American College of Cardiology, 51, 1357–1365. 18387436 10.1016/j.jacc.2007.09.073

[anec13056-bib-0009] Dunn, A. J. , ElRefai, M. H. , Roberts, P. R. , Coniglio, S. , Wiles, B. M. , & Zemkoho, A. B. (2021). Deep learning methods for screening patients' S‐ICD implantation eligibility. Artificial Intelligence in Medicine, 119, 102139. 10.1016/J.ARTMED.2021.102139 34531008

[anec13056-bib-0010] Fan, X. , Yao, Q. , Cai, Y. , Miao, F. , Sun, F. , & Li, Y. (2018). Multiscaled fusion of deep convolutional neural networks for screening atrial fibrillation from single Lead short ECG recordings. IEEE Journal of Biomedical and Health Informatics, 22(6), 1744–1753. 10.1109/JBHI.2018.2858789 30106699

[anec13056-bib-0011] Francia, P. , Ziacchi, M. , de Filippo, P. , Viani, S. , D'Onofrio, A. , Russo, V. , Adduci, C. , Biffi, M. , Ferrari, P. , Bianchi, V. , Ammendola, E. , Palano, F. , Frisoni, J. , Valsecchi, S. , Lovecchio, M. , & Bongiorni, M. G. (2018). Subcutaneous implantable cardioverter defibrillator eligibility according to a novel automated screening tool and agreement with the standard manual electrocardiographic morphology tool. Journal of Interventional Cardiac Electrophysiology, 52(1), 61–67. 10.1007/S10840-018-0326-2 29502193

[anec13056-bib-0012] Groh, C. A. , Sharma, S. , Pelchovitz, D. J. , Bhave, P. D. , Rhyner, J. , Verma, N. , Arora, R. , Chicos, A. B. , Kim, S. S. , Lin, A. C. , Passman, R. S. , & Knight, B. P. (2014). Use of an electrocardiographic screening tool to determine candidacy for a subcutaneous implantable cardioverter‐defibrillator. Heart Rhythm, 11(8), 1361–1366. 10.1016/J.HRTHM.2014.04.025 24755323

[anec13056-bib-0013] Hasan, M. A. , Abbott, D. , & Baumert, M. (2012). Relation between beat‐to‐beat qt interval variability and t‐wave amplitude in healthy subjects. Annals of Noninvasive Electrocardiology., 17(3), 195–203. 10.1111/j.1542-474X.2012.00508.x 22816538 PMC6932369

[anec13056-bib-0015] Jarman, J. W. E. , Lascelles, K. , Wong, T. , Markides, V. , Clague, J. R. , & Till, J. (2012). Clinical experience of entirely subcutaneous implantable cardioverter‐defibrillators in children and adults: Cause for caution. European Heart Journal, 33, 1351–1359. 22408031 10.1093/eurheartj/ehs017

[anec13056-bib-0016] Kamp, N. J. , & Al‐Khatib, S. M. (2019). The subcutaneous implantable cardioverter‐defibrillator in review. American Heart Journal, 217, 131–139. 10.1016/j.ahj.2019.08.010 31654943

[anec13056-bib-0017] Kiranyaz, S. , Ince, T. , & Gabbouj, M. (2016). Real‐time patient‐specific ECG classification by 1‐D convolutional neural networks. IEEE Transactions on Biomedical Engineering, 63(3), 664–675. 10.1109/TBME.2015.2468589 26285054

[anec13056-bib-0018] Knops, R. E. , Olde Nordkamp, L. R. A. , Delnoy, P. P. H. M. , Boersma, L. V. A. , Kuschyk, J. , el‐Chami, M. F. , Bonnemeier, H. , Behr, E. R. , Brouwer, T. F. , Kääb, S. , Mittal, S. , Quast, A. F. B. E. , Smeding, L. , van der Stuijt, W. , de Weger, A. , de Wilde, K. C. , Bijsterveld, N. R. , Richter, S. , Brouwer, M. A. , … Wilde, A. A. M. (2020). Subcutaneous or transvenous defibrillator therapy. New England Journal of Medicine, 383(6), 526–536. 10.1056/NEJMoa1915932 32757521

[anec13056-bib-0019] Lambiase, P. D. , Theuns, D. A. , Murgatroyd, F. , Barr, C. , Eckardt, L. , Neuzil, P. , Scholten, M. , Hood, M. , Kuschyk, J. , Brisben, A. J. , Carter, N. , Stivland, T. M. , Knops, R. , LVA, B. , & Investigators on behalf of the E . (2022). Subcutaneous implantable cardioverter‐defibrillators: Long‐term results of the EFFORTLESS study. European Heart Journal, 43, 1–14. 10.1093/EURHEARTJ/EHAB921 PMC9156377 35090007

[anec13056-bib-0020] Lih, O. S. , Jahmunah, V. , San, T. R. , Ciaccio, E. J. , Yamakawa, T. , Tanabe, M. , Kobayashi, M. , Faust, O. , & Acharya, U. R. (2020). Comprehensive electrocardiographic diagnosis based on deep learning. Artificial Intelligence in Medicine, 103, 103. 10.1016/j.artmed.2019.101789 32143796

[anec13056-bib-0021] Liu, W. , Zhang, M. , Zhang, Y. , Liao, Y. , Huang, Q. , Chang, S. , Wang, H. , & He, J. (2018). Real‐time multilead convolutional neural network for myocardial infarction detection. IEEE Journal of Biomedical and Health Informatics, 22(5), 1434–1444. 10.1109/JBHI.2017.2771768 29990164

[anec13056-bib-0022] Madias, J. E. (2005). QTc interval in patients with changing edematous states: Implications on interpreting repeat QTc interval measurements in patients with anasarca of varying etiology and those undergoing hemodialysis. PACE ‐ Pacing and Clinical Electrophysiology., 28(1), 54–61. 10.1111/j.1540-8159.2005.09384.x 15660804

[anec13056-bib-0023] Madias, J. E. , Bazaz, R. , Agarwal, H. , Win, M. , & Medepalli, L. (2001). Anasarca‐mediated attenuation of the amplitude of electrocardiogram complexes: A description of a heretofore unrecognized phenomenon. Journal of the American College of Cardiology, 38(3), 756–764. 11527629 10.1016/s0735-1097(01)01429-2

[anec13056-bib-0024] Maurizi, N. , Olivotto, I. , Olde Nordkamp, L. R. A. , Baldini, K. , Fumagalli, C. , Brouwer, T. F. , Knops, R. E. , & Cecchi, F. (2016). Prevalence of subcutaneous implantable cardioverter‐defibrillator candidacy based on template ECG screening in patients with hypertrophic cardiomyopathy. Heart Rhythm., 13(2), 457–463. 10.1016/J.HRTHM.2015.09.007 26362577

[anec13056-bib-0025] Mayuga, K. A. , & Fouad‐Tarazi, F. (2007). Dynamic changes in T‐wave amplitude during tilt table testing: Correlation with outcomes. Annals of Noninvasive Electrocardiology, 12(3), 246–250. 10.1111/J.1542-474X.2007.00168.X 17617070 PMC6932404

[anec13056-bib-0026] Miao, F. , Wen, B. , Hu, Z. , Fortino, G. , Wang, X. P. , Liu, Z. D. , Tang, M. , & Li, Y. (2020). Continuous blood pressure measurement from one‐channel electrocardiogram signal using deep‐learning techniques. Artificial Intelligence in Medicine, 108, 108. 10.1016/j.artmed.2020.101919 32972654

[anec13056-bib-0027] Olde Nordkamp, L. R. A. , Dabiri Abkenari, L. , Boersma, L. V. A. , Maass, A. H. , de Groot, J. R. , van Oostrom, A. J. H. H. M. , Theuns, D. A. M. J. , Jordaens, L. J. L. M. , Wilde, A. A. M. , & Knops, R. E. (2012). The entirely subcutaneous implantable cardioverter‐defibrillator. Journal of the American College of Cardiology, 60, 1933–1939. 23062537 10.1016/j.jacc.2012.06.053

[anec13056-bib-0028] Olde Nordkamp, L. R. A. , Warnaars, J. L. F. , Kooiman, K. M. , de Groot, J. R. , BRAM, R. , AAM, W. , & Knops, R. E. (2014). Which patients are not suitable for a subcutaneous ICD: Incidence and predictors of failed QRS‐T‐wave morphology screening. Journal of Cardiovascular Electrophysiology, 25(5), 494–499. 10.1111/JCE.12343 24320684

[anec13056-bib-0029] Pourbabaee, B. , Roshtkhari, M. J. , & Khorasani, K. (2018). Deep convolutional neural networks and learning ECG features for screening paroxysmal atrial fibrillation patients. IEEE Transactions on Systems, Man, and Cybernetics: Systems, 48(12), 2095–2104. 10.1109/TSMC.2017.2705582

[anec13056-bib-0030] Randles, D. A. , Hawkins, N. M. , Shaw, M. , Patwala, A. Y. , Pettit, S. J. , & Wright, D. J. (2014). How many patients fulfil the surface electrocardiogram criteria for subcutaneous implantable cardioverter‐defibrillator implantation? EP Europace, 16(7), 1015–1021. 10.1093/EUROPACE/EUT370 24351884

[anec13056-bib-0014] Roberts, F. M. , Povinelli, R. J. , & Ropella, K. M. (2001). Identification of ECG arrhythmias using phase space reconstruction. In L. De Raedt & A. Siebes (Eds.), Principles of Data Mining and Knowledge Discovery. PKDD 2001. Lecture Notes in Computer Science (Vol. 2168). Springer. 10.1007/3-540-44794-6_34

[anec13056-bib-0031] Rocha, T. , Paredes, S. , de Carvalho, P. , Henriques, J. , & Antunes, M. (2008). Phase space reconstruction approach for ventricular arrhythmias characterization. Proceedings of the 30th Annual International Conference of the IEEE Engineering in Medicine and Biology Society. EMBS'08 ‐ “Personalized Healthcare through Technology, 2008, 5470–5473. 10.1109/IEMBS.2008.4650452 19163955

[anec13056-bib-0032] Rudic, B. , Tulumen, E. , & Fastenrath, F. (2018). P917Evaluation of a new automated screening tool for the assessment of the eligibility for a subcutaneous implantable‐cardioverter defibrillator. EP Europace, 20(suppl_1), i177–i178. 10.1093/EUROPACE/EUY015.518

[anec13056-bib-0033] Sangaiah, A. K. , Arumugam, M. , & Bin, B. G. (2020). An intelligent learning approach for improving ECG signal classification and arrhythmia analysis. Artificial Intelligence in Medicine, 103, 101788. 10.1016/j.artmed.2019.101788 32143795

[anec13056-bib-0034] Srinivasan, N. T. , Patel, K. H. , Qamar, K. , Taylor, A. , Bacà, M. , Providência, R. , Tome‐Esteban, M. , Elliott, P. M. , & Lambiase, P. D. (2017). Disease severity and exercise testing reduce subcutaneous implantable cardioverter‐defibrillator left sternal ECG screening success in hypertrophic cardiomyopathy. Circulation: Arrhythmia and Electrophysiology, 10(4), e004801. 10.1161/CIRCEP.117.004801 28408651

[anec13056-bib-0035] Vemishetty, N. , Gunukula, R. L. , Acharyya, A. , Puddu, P. E. , Das, S. , & Maharatna, K. (2019). Phase space reconstruction based CVD classifier using localized features. Scientific Reports, 9(1), 1–18. 10.1038/s41598-019-51,061-8 31601877 PMC6787214

[anec13056-bib-0036] Walker, B. D. , Krahn, A. D. , Klein, G. J. , Skanes, A. C. , & Yee, R. (2003). Drug induced QT prolongation: Lessons from congenital and acquired long QT syndromes. Current Drug Targets. Cardiovascular & Haematological Disorders, 3(4), 327–335. 10.2174/1568006033481393 14683474

[anec13056-bib-0037] Wiles, B. M. , Morgan, J. M. , Allavatam, V. , ElRefai, M. , & Roberts, P. R. (2021). S‐ICD screening revisited: Do passing vectors sometimes fail? Pacing and Clinical Electrophysiology. Published online December, 26, 182–187. 10.1111/PACE.14424 34881431

[anec13056-bib-0038] Zhang, J. , Liu, A. , Gao, M. , Chen, X. , Zhang, X. , & Chen, X. (2020). ECG‐based multi‐class arrhythmia detection using spatio‐temporal attention‐based convolutional recurrent neural network. Artificial Intelligence in Medicine, 106, 106. 10.1016/j.artmed.2020.101856 32593390

